# Depression and Hand-Grip: Unraveling the Association

**DOI:** 10.7759/cureus.38632

**Published:** 2023-05-06

**Authors:** Vijay Durga Pradeep Ganipineni, Ajay Sai Krishna Kumar Idavalapati, Samuel Sowrab Tamalapakula, Vagdevi Moparthi, Monica Potru, Oluwasayo J Owolabi

**Affiliations:** 1 Department of General Medicine, SRM Medical College Hospital and Research Center, Chennai, IND; 2 Department of General Medicine, Andhra Medical College/King George Hospital, Visakhapatnam, IND; 3 Department of Medicine, Guntur Medical College, Guntur, IND; 4 Department of Medicine, Katuri Medical College and Hospital, Guntur, IND; 5 Department of Medicine, Dr. Pinnamaneni Siddhartha Institute of Medical Sciences and Research Foundation, Vijayawada, IND; 6 Department of Medicine, Guntur Medial College, Guntur, IND; 7 Department of Psychiatry, Lugansk State Medical University, Lugansk, UKR

**Keywords:** grip force, hospital anxiety depression scale (hads), : sarcopenia, muscle strength, hand grip dynamometer, hand grip, mdd, major depressive disorder, depression, mental health assessment

## Abstract

This review article explores the association between hand-grip strength and depression. A total of 14 studies were carefully considered to provide a comprehensive analysis of the topic. The studies reveal a consistent association between low hand-grip strength and depressive symptoms, independent of age, gender, and chronic disease status. The evidence suggests that hand-grip strength assessment could be a useful tool for identifying individuals at risk of depression, particularly older adults and those with chronic diseases. Incorporating physical activity and strength training into treatment plans can contribute to better mental health outcomes. Hand-grip strength assessment can also be used as a monitoring tool to track changes in physical and mental health over time in individuals with depression. Healthcare professionals should consider the relationship between hand-grip strength and depression when evaluating patients and developing treatment plans. The findings from this comprehensive clinical review have important clinical implications and highlight the importance of considering physical health factors in the context of mental health.

## Introduction and background

Hand-grip strength, a simple yet powerful measure of overall muscular strength, has emerged as a topic of growing interest within the healthcare community [1]. As an easily accessible and reliable measure, it has garnered attention for its potential implications in a wide array of clinical domains. One of the most intriguing areas of investigation is the association between hand-grip strength and depression, a debilitating mental health disorder that affects millions of individuals worldwide [2].

Depression, characterized by persistent feelings of sadness, hopelessness, and a lack of interest or pleasure in activities, is a leading cause of disability and suffering across the globe [3]. The World Health Organization estimates that more than 280 million people of all ages suffer from depression, making it one of the most common mental health disorders [3]. The significant personal, societal, and economic impacts of depression cannot be overstated, highlighting the urgent need for effective prevention and treatment strategies [4].

One of the most challenging aspects of depression is its complex etiology, which involves a combination of genetic, environmental, and psychological factors [4]. This complexity necessitates a multifaceted approach to understanding and addressing the disorder, with a focus on identifying modifiable risk factors that can be targeted through preventive and therapeutic interventions [5]. Among the myriad factors implicated in the development and progression of depression, physical health indicators such as hand-grip strength have emerged as a promising area of research [5].

Hand-grip strength, which is strongly correlated with systemic muscle strength, is one of the promising candidates for the brief evaluation of physical function. Moreover, hand-grip strength is also used to predict future activities of daily life [6,7]. Hand-grip strength is an easily accessible, non-invasive, and cost-effective measure of muscular strength, making it an attractive candidate for clinical research and practice [6,7]. It has been shown to correlate with overall physical health, functional capacity, and even mortality, emphasizing its potential relevance to a broad spectrum of health outcomes [5]. Moreover, the assessment of hand-grip strength can be conducted with minimal equipment and training, making it an ideal tool for both research and clinical settings.

Given the known benefits of physical activity and exercise in promoting mental health and well-being, it is perhaps unsurprising that a growing body of evidence has begun to explore the relationship between hand-grip strength and depression [8]. Several studies have reported associations between low hand-grip strength and increased depressive symptoms, suggesting that this simple measure may hold the key to understanding and addressing the complex interplay between physical and mental health [9].

However, the existing literature on the association between hand-grip strength and depression is characterized by a degree of heterogeneity in terms of study populations, methodologies, and outcomes. This diversity has generated both complementary and conflicting findings, underscoring the need for a comprehensive synthesis of the available evidence. By reviewing and integrating the findings from 14 key studies, this clinical review endeavors to provide a more coherent and nuanced understanding of the relationship between hand-grip strength and depression and elucidate the potential clinical implications of this association.

Methods

A literature search was conducted to identify relevant studies investigating the association between hand-grip strength and depression from inception till date. Major databases, including PubMed, Google Scholar and Central, were diligently searched by including studies conducted on humans and published in English. The keywords used in the search included "hand grip strength," "muscle strength," "depression," “mdd,” "mood disorder," "mental health," and "psychological well-being." The keywords were used in various combinations and searched using Boolean operators (AND, OR) to ensure a comprehensive search. Fourteen studies were carefully considered and analyzed in this review. These studies encompassed a range of study designs, including cross-sectional, longitudinal, and case-control studies, and were conducted in various countries and population groups.

Data were extracted from each study, including study design, sample characteristics (age distribution and population setting), depression assessment tools, and key findings. The extracted data were organized into a table, summarizing the essential features and results of each study. The study findings were synthesized to provide a comprehensive understanding of the association between hand-grip strength and depression.

The review focused on examining the consistency and variability of findings across studies, considering factors such as age, gender, and chronic disease status that may influence the relationship between hand-grip strength and depression. Additionally, the review assessed potential confounding factors and clinical implications of the study findings.

## Review

Fourteen studies were analyzed in this review, including Sternäng et al. 2016 [10], Ashdown-Franks et al. 2018 [11], Musalek et al. 2017 [12], Smith et al. 2022 [13], Mergl et al. 2007 [14], Zheng et al. 2022 [15], Kim et al. 2019 [2], Gu et al. 2021 [16], Phillips et al. 2011 [17], Marconcin et al. 2020 [18], McDowell et al. 2018 [19], Fukumori et al. 2015 [9], Smith et al. 2019 [20], Brook et al. 2018 [21], to provide a balanced synopsis of the contemporary literature.

The association between hand-grip strength and depression has been investigated in various populations, age groups, and settings. The 14 included studies reviewed here (Table 1) demonstrate that the relationship between hand-grip strength and depression is complex, with some studies showing a negative association between grip strength and depressive symptoms, while others have found no significant relationship or differential effects based on factors such as age, gender, or chronic disease status.

**Table 1:** Summary of characteristics of included studies CESDS: 8-item Center for Epidemiologic Studies Depression Scale, PHQ-9: Patient Health Questionnaire, BDI-II: Beckers Depression Inventory, HDR: Hamilton Depression Rating Scale, SDS: Self-Rated Depression Scale, MHI-5: Mental Health Inventory, EURO-D: European Depression Symptoms 12-item scale

Study ID	Type of Study	Age	Handgrip Assessment	Depression Questionnaire	Conclusion
Sternäng et al. 2016 [10]	Longitudinal Cohort study	40-86	Collins hand grip dynamometer	N/A	Grip strength is inversely related to the risk of cognitive decline.
Ashdown-Franks. 2021 [11]	Cross-sectional study	>50		CIDI	Grip strength is negatively associated with depression severity.
Musalek et al. 2017 [12]	Cross-sectional study	18-30	Hand-hold calibrated dynamometer (JAMAR, Hatfield, PA, USA)	BDI-II	No significant relationship between grip strength and depressive symptoms.
Smith et al. 2022 [13]	Longitudinal Cohort study	Adults (mean age: 63.7)	Dynamometer	PHQ-9	Greater handgrip strength is associated with lower risk of new-onset depressive symptoms.
Mergl et al. 2007 [14]	Longitudinal Cohort study. Two-part study.	Not specified	Digitizing graphic tablet and kinematic analysis of handwriting and rapid drawing movements.	HDR	Depressed patients show irregular hand-motor patterns; differential pharmaceutical effects on hand-motor function.
Zheng et al. 2022 [15]	Longitudinal cohort study	50+	Smedley handheld dynamometer (100 kg)	EURO-D	Interaction between grip strength and physical activity on depression; lower grip strength and inactivity worsen depression.
Kim et al. 2019 [2]	Longitudinal cohort study	Middle-aged and elderly	Handgrip dynamometer (Model number: NO6103, Manufacturer: TANITA, Japan)	MMSE, CES-D	Negative association between grip strength and cognitive decline and depressive symptoms.
Gu et al. 2021 [16]	Cross-sectional study	41.5 ± 11.9	Dynamometer	SDS	Inverse association between grip strength and depressive symptoms, stronger in females.
Phillips et al. 2011 [17]	Case-control study	Not specified	JAMAR 5-position grip strength dynamometer (Sammons Preston, Bolingbrook, IL)	DSM-IV	Clinically depressed individuals may produce results suggesting poor volitional effort in 5-position grip strength testing.
Marconcin et al. 2020 [18]	Cross-sectional study	Middle-aged and older adults	Smedley handgrip dynamometer	EURO-D	Association between grip strength and depressive symptoms depends on chronic conditions, age group, and gender.
McDowell et al. 2018 [19]	Cross-sectional and longitudinal cohort study	50+	Hand-held dynamometer	CESDS	Inverse association between grip strength and incident depression in older adults, stronger in females.
Fukumori et al. 2015 [9]	Longitudinal cohort study	40-79	Digital dynamometer	MHI-5	Lower hand-grip strength is both cross-sectionally and longitudinally associated with depressive symptoms.
Smith et al. 2019 [20]	Cross-sectional study	50+	Handheld dynamometer	CESDS	Grip strength was negatively associated with depressive symptoms in older adults in England.
Brooks et al. 2018 [21]	Cross-sectional study	60+	Handheld dynamometer	PHQ-9	Reduced levels of combined hand-grip strength are independently associated with greater depressive symptoms among U.S. adults aged 60 years and older.

A majority of the studies, including longitudinal and cross-sectional designs, found an inverse relationship between grip strength and depression, with greater grip strength associated with lower depression risk or severity (Ashdown-Franks et al. [11], Smith et al. [13], Zheng et al. [15], Kim et al. [2], Gu et al. [16], Marconcin et al. [18], McDowell et al. [19], Fukumori et al. [9]). This association was observed in various age groups, from middle-aged to elderly populations.

Some studies noted gender differences in the relationship between grip strength and depression (Gu et al. [16], McDowell et al. [19]). In these cases, stronger associations were observed among females compared to males. Additionally, Marconcin et al. [18] highlighted that the association between grip strength and depressive symptoms should consider factors such as chronic conditions, age group, and gender.

However, Musalek et al. [12] found no significant relationship between grip strength and depressive symptoms in a younger population aged 18-30. Furthermore, Mergl et al. [14] and Phillips et al. [17] focused on motor patterns and grip strength test interpretation in depressed patients, raising questions about the potential confounding effect of depression on the assessment of hand-grip strength.

In a groundbreaking study, Zhang et al. [22] explored the shared genetic variation between depression and grip strength using twin data from Qingdao, China. They discovered potential genetic correlations, Single Nucleotide Polymorphisms (SNPs), genes, and pathways common to both conditions [22]. The study revealed a moderate genetic correlation between grip strength and depression, with a genetic correlation coefficient of -0.41 (-0.96, -0.15), indicating a shared genetic basis [22]. Key findings in the SNP-based analysis included rs117744620, located in the LRR1 gene on chromosome 14, which exceeded the genome-wide significance level. LRR1 is involved in regulating 4-1BB-mediated signaling cascades that activate NF-кB [23], which could impact depression. LRR1 can also affect actin [24], thereby influencing muscle strength. Three SNPs (rs117546604, rs150220336, rs147079354) found in or near the ME2 gene also exceeded the genome-wide significance level. ME2, which encodes a mitochondrial NAD-dependent malic enzyme, has been linked to generalized epilepsy [25], psychosis, mania [26], and PI3K/AKT signaling [27], suggesting its association with both the nervous system and muscle strength as well as depression.

Among the top 60 SNPs with suggested levels of association, three SNPs (rs75534602, rs117533783, rs78161270) were located near the DDC gene [22]. The DDC gene has been linked to postpartum anxiety [28] and aromatic L-amino acid decarboxylase deficiency (AADCD), which include early-onset hypotonia [29] as a primary symptom. This suggests that the DDC gene might simultaneously affect grip strength and depression. Other notable genes include P2RX7, involved in inflammatory responses and neuroimmune mechanisms, and PRKD1, which regulates actin [30] and is associated with neurons [31], NF-кB [32], and the inflammatory response [33].

In gene-based analysis, two significant genes, GTF2H2C_2 and GTF2H2C were identified, but their relationship with the phenotype remains unclear due to insufficient research [22]. Several genes with nominal significance levels were found to be associated with grip strength and depression, such as FNBP1, MOG, SACS, KMO, TECPR2, and MGTA5 [22]. Additionally, expression levels of KCNN4, MOG, and SNHG12 genes can influence inflammatory factors [34-39], potentially impacting grip strength and depression through their effects on the inflammatory response [22].

Pathway enrichment analysis revealed numerous pathways related to hormone synthesis and neural signal transduction, such as those involving androgen biosynthesis, steroid hormones and potassium channels, a deficiency of which is known to cause both muscle weakness and depression [40-43]. Additionally, pathways, including RHO GTPases, fibroblast growth factors, and cytokine receptor interactions may collectively influence grip strength and depression through various mechanisms, actin regulation [44], neuronal development [45,46], skeletal muscle development [47], and inflammatory responses.

Brooks et al. [21] performed a cross-sectional study utilizing the NHANES 2011-2014 data and reported that reduced levels of combined handgrip strength are independently associated with greater depressive symptoms among U.S. adults aged 60 years and older. They found that this significance level is only found in the age group 60-69 years, owing to the difficulty in adjusting to senility.

Clinical implications

The findings gleaned from this clinical review give rise to several vital clinical implications that warrant further discussion. It has been postulated that a biological connection, such as hormonal imbalances or changes in neurotransmitter levels in individuals with depression, might impact muscle function and consequently result in decreased handgrip strength [22]. Furthermore, chronic inflammation, which has been linked to both depression and reduced muscle strength, could play a role in the observed association between the two factors [48,49].

Another consideration is the influence of physical activity levels on the relationship between depression and handgrip strength. It is well-established that individuals with depression often experience reduced motivation and energy levels, leading to decreased physical activity, which may contribute to a decline in muscle strength, including handgrip strength [21]. Likewise, poor nutrition or appetite changes common in people with depression could lead to muscle wasting and decreased handgrip strength [50].

Sleep disturbances, frequently observed in individuals with depression, can negatively impact muscle recovery and strength [51]. The association between handgrip strength and depression may indicate that people with depression have a lower overall health status, as handgrip strength has been considered a marker of overall health and physical fitness [52]. Additionally, depression's negative impact on cognitive function, including attention and concentration, may contribute to reduced handgrip strength [53].

The association between handgrip strength and depression could provide a simple, non-invasive tool for early detection and intervention in individuals at risk for depression. Handgrip strength measurement could be included in routine health assessments to identify those who may benefit from further mental health evaluation. Moreover, the findings could have implications for the development of new treatment approaches that target both depression and physical fitness, such as exercise-based interventions or combining psychotherapy with physical rehabilitation programs.

Public health campaigns and policies aimed at promoting mental and physical well-being could also be informed by these study results, emphasizing the importance of regular physical activity and mental health support for the general population. Furthermore, the study suggests potential shared genetic factors and pathways involved in both depression and handgrip strength [22], which could have implications for future research into the biological underpinnings of these conditions and potential treatment targets.

The interplay between physical and mental health is underscored by these findings, as depression is often associated with physical symptoms, such as fatigue and muscle weakness, and impaired handgrip strength could be a manifestation of these symptoms [54]. Alternatively, poor handgrip strength could contribute to a sense of physical limitation and low mood. Screening for depression and handgrip strength may be useful in identifying individuals at risk for these conditions, and interventions aimed at promoting physical activity and strength training could have potential benefits for both physical and mental health.

Finally, the study's findings could have implications for clinical practice, particularly in the context of geriatric care. Handgrip strength is often used as a measure of frailty in older adults and has been linked to a range of negative health outcomes [55]. The association between depression and grip strength could be an important consideration in the assessment and management of older adults' health.

Future directions

The findings from the studies encompassed in this review consistently indicate a significant negative association between handgrip strength and depression. The groundbreaking research by Zhang et al. [22] uncovered shared genetic factors that may contribute to the association between grip strength and depression, providing valuable insight into the potential biological underpinnings of this relationship. Further research is warranted to deepen our understanding of the mechanisms underlying the association between grip strength and depression, including the potential role of genetics, the impact of confounding factors, and the influence of gender, age, and chronic disease status on this relationship. The results also pave the way for further exploration into the genetic and pathophysiological foundations of mental and physical health. We hope that such investigations will uncover innovative therapeutic targets for addressing both depression and physical performance, ultimately enhancing the overall well-being of individuals.

Limitations

First, it is essential to recognize the methodological differences among the studies. The studies included a mix of cross-sectional, cohort, and case-control designs, which provide varying levels of evidence regarding causality. Longitudinal studies, such as those by Fukumori et al. [9] and McDowell et al. [19], offer stronger evidence for the potential causal relationship between hand-grip strength and depression. However, the majority of the studies included in this review were cross-sectional, limiting our ability to draw definitive conclusions about the directionality of the relationship between hand-grip strength and depression.

The studies also employed different measures of depression, such as the Center for Epidemiologic Studies Depression Scale (CESDS), the Mental Health Inventory (MHI-5), and the European Depression Symptoms 12-item scale (EURO-D). These variations in measurement may contribute to inconsistencies in the findings, making it challenging to compare results across studies directly.

Despite these methodological differences, several consistent findings emerged from the 14 studies. Most notably, a majority of the studies reported a significant association between lower hand-grip strength and increased depressive symptoms or depression risk, independent of age and gender. This association was observed in various populations, including older adults, individuals with chronic diseases, and community-dwelling adults.

However, it is crucial to consider the role of potential confounding factors in the relationship between hand-grip strength and depression. For instance, Marconcin et al. [18] found that the association between grip strength and depressive symptoms varied based on the presence of chronic diseases. The study observed a significant relationship among participants with no chronic diseases and those with metabolic diseases but not among those with arthritis diseases [18]. This finding underscores the importance of considering the individual's specific health status when evaluating the association between hand-grip strength and depression.

Furthermore, some studies identified potential sex differences in the relationship between hand-grip strength and depression. For example, McDowell et al. [19] found that grip strength was inversely associated with incident depression in older adults, with stronger associations observed among females than males. This finding suggests that the relationship between hand-grip strength and depression may be influenced by sex-specific factors, which warrants further investigation.

In addition to these consistent findings, several studies also reported more nuanced results that could inform future research and clinical practice. For example, Phillips et al. [17] found that individuals with clinical depression were more likely to produce grip strength measures suggestive of poor volitional effort, which could be misinterpreted by clinicians as a lack of sincerity of effort. This finding highlights the importance of considering the potential impact of depression on grip strength testing and the need for healthcare professionals to be aware of this potential confounding factor.

In summary, these studies provide valuable insights into the association between hand-grip strength and depression. While the methodological differences and potential confounding factors limit our ability to draw definitive conclusions, the consistent findings across studies suggest that there is a significant relationship between lower hand-grip strength and increased depressive symptoms or depression risk. This relationship appears to be influenced by factors such as age, gender, and chronic disease status, highlighting the need for a personalized approach to understanding and addressing the complex interplay between hand-grip strength and depression.

Future research should focus on addressing the methodological limitations of the existing literature, including the need for more longitudinal studies to establish causality, as well as the need for standardized depression measurement tools to facilitate comparisons across studies. Moreover, additional research should investigate the underlying mechanisms that link hand-grip strength and depression, as well as the potential moderating factors, such as age, sex, and chronic disease status, which may influence this relationship.

## Conclusions

This review of literature on the association between hand-grip strength and depression offers valuable insights into the complex relationship between these factors. Although methodological differences and potential confounding factors limit our ability to draw definitive conclusions, the consistent findings across studies suggest a significant negative association between hand-grip strength and depression. This relationship appears to be influenced by factors such as age, gender, and chronic disease status, underlining the importance of a personalized approach to understanding and addressing this intricate interplay.

The interplay between physical and mental health is underscored by these findings, emphasizing the need for future research to investigate the underlying mechanisms linking hand-grip strength and depression and potential moderating factors. Longitudinal studies and standardized depression measurement tools are essential for facilitating comparisons across studies and establishing causality. Furthermore, these findings highlight the potential for hand-grip strength measurement to serve as a simple, non-invasive tool for early detection and intervention in individuals at risk for depression. The results of this review have significant implications for clinical practice, public health campaigns, and policies aimed at promoting mental and physical well-being. Interventions that target both depression and physical fitness, such as exercise-based interventions or combining psychotherapy with physical rehabilitation programs, could have potential benefits for both physical and mental health. Additionally, the findings could inform the development of new treatment approaches focusing on shared genetic factors and pathways involved in both depression and hand-grip strength.
